# The Vegetable Kingdom; or the Structure, Classification, and Uses of Plants, Illustrated upon the Natural System

**Published:** 1847-04-01

**Authors:** 


I. The Vegetable Kingdom; or the Structure, Classification and Uses of Plants, illustrated upon the Natural System. By *John Lindley*, Ph. D. F.R.S. & F.L.S. Professor of Botany in the University of London and in the Royal Institution of Great Britain. With upwards of Five Hundred Illustrations. 8vo. pp. 911. London, 1846.

II. Outlines of Structural and Physiological Botany. By *Arthur Henfrey*, F.L.S. Lecturer on Botany at the Middlesex Hospital, late Botanist to the Geological Survey of the United Kingdom. With numerous illustrations. In three Parts. Part One—Elementary Structures. Part Two—Organs of Vegetation. Pp. 106. London.

There is not one of the collateral departments of medicine which has presented a more altered appearance within the last fifteen or twenty years than the science of Botany, whether we regard the advances made in the establishment of philosophical and fixed principles to reason upon, or the manner in which the student of our art is initiated into a knowledge of the fundamental doctrines of this important and interesting hand-maid of medicine. The time has not long passed since the study of botany, whether pursued by the professional student or the cultivator of general science, was a study of classification and of terms, and it might well be argued that, as manifold and imperious were the strictly practical duties of a professional career, it was but of slight importance, whether during our noviciate we paid much attention to mere catalogues and words. Now, however, the whole face of matters has changed; instead of the different kingdoms of Nature being severed from each other, each broken up again into minute portions, and regarded only in a few and comparatively unimportant relationships, the study of the organized creation has been viewed as a whole, the relations of development between apparently distinct portions shewn, the analogies of structure and function, not only between particular groups of vegetable organisms beautifully evolved, but, even with the apparently opposed forms of animal existence, remarkable coincidence of obedience to the same laws of evolution demonstrated to exist.

The same philosophical principles of investigation which have been applied to the study of man and animals are now applied to that of plants. As there is *General and Special Anatomy* of the former, so there is of the latter; as there is *History of Function* of the one, so is there of the other. There proceed the functions of *Digestion* and *Respiration* in vegetable bodies as do these actions in other organisms, and the processes of *Impregnation* and *Development* are as complex in the one as in the other. It is true that, as yet, no analogues of the nervous centres or system have been satisfactorily demonstrated to exist in plants, but even in respect to this important endowment of the higher forms of organized creation, there are some writers who have expressed their belief of its existence, however modified it may be, in vegetable beings. No doubt can now exist that, he who wishes to have a right knowledge of the laws of life, and a due notion of what is denominated *General Physiology*, must apply his attention equally to plants and to animals. The laws of *primary development* form leading features of modern advanced physiology, and, as they are seen exhibited in both kingdoms of Nature, they must of necessity be studied. In such study it will be found that, the laws of the one are nearly identical with those of the other, and that, however the more complex combinations which arise from the formative powers of the different races of beings at one part of the scale differ, yet originally all the forms of organized matter are made up of a series of minute structural elements which are identical in the two departments of Nature. Some persons have at times gone much further, or, as Mr. Henfrey remarks in his Introduction,

"Some philosophers have even denied the possibility of defining the limits between the organic and the inorganic world—between a cell and a crystal. Without admitting the validity of this view, which, although plausible in theoretical expositions, is practically negatived by very slight investigation, it must be allowed that the axiom *natura non facit saltus*, holds good so far as relates to the organic creation; the animal and vegetable kingdoms, so distinct and opposite both in appearance and function, when taken in their entirety, approach gradually by their lower tribes, until at the extreme point they meet in a simple cell which stands as it were on the limit."

In former days, the lecturer on botany generally commenced his duties by giving his students definitions of terms, then followed an exposition of the artificial system of Linnæus, and these, varied with a description of different forms of roots, kinds of leaves and flowers, composed the staple of the learner's induction into "the lore of the green herb and the bee-worshipped flower." Now, he unfolds to them comprehensive views of the development of elementary tissues, of the varied combinations of secondary structures arising from these, in fact they are as well grounded in the structural anatomy of a plant, as they are intended to be in the knowledge of that of man, and are shown the intimate relationship existing between the one and the other. Then follows the consideration of structure in action, in other words, the history of function is gone into; and, finally, the student has laid before him the advantages taken from known identities of structure, function and property, to arrange together the various denizens of the vegetable world in systematic groups, and the more important members of these groups as bearing a relation to medicine are described to him in detail. If, in considering the former two departments of the science of botany, he sees exhibited the close and intimate connexion between them and like departments of the history of animal life, in the latter he is no less struck with the valuable results which have accrued to the practical portion of his art—materia medica. How, by reasoning upon the law that, with like structure there is like property, he may avoid, in a strange climate, administering a hitherto to him unknown vegetable production, because the plant that yields it is connected by affinities to others in his own climate, which he has learnt to be of a deleterious nature. Or conversely how he may safely add to the available treasures he already possesses, the product of a plant which otherwise—were he ignorant of its congeners—he might be timid in employing. The student will find, to use the words of the author of the admirable work which is placed at the head of our notice, in another place, that the science of botany, as it should now be taught him. "is the science which converts the useless or noxious weed into the nutritious vegetable, which changes a bare volcanic rock into a green and fertile island, and which enables the man of science, by the power it gives him of studying how far the productions of one climate are susceptible of cultivation in another, to guide the colonist in his enterprises, and to save him from those errors and losses into which all such persons unacquainted with botany are liable to fall. This science finally it is which teaches the physician how to discover in every region the medicines that are best adapted for the maladies prevalent in it, and which, by furnishing him with a certain clue to the knowledge of the tribes in which particular properties are or are not to be found, renders him as much at ease alone, and seemingly without resources, in a land of unknown herbs, as if he were in the midst of a magazine of drugs in some civilized country."—(*Lindley's Introduction to Botany*).

In illustration of some of those circumstances which have so helped to change the aspect of botanical science and vegetable physiology within the last few years, we may remark, that a claim of first importance is to be allotted to the discovery and elucidation of the theory of what is termed *cell development*. The researches of Schwann, Schleiden, Karl Müller, Purkinjie, Valentin and others, not only placed the mode of development and form of evolution of the basic structure of vegetable organisms in their true light, but connected that development and evolution in the lower forms of life by a rich series of microscopic demonstrations with that of the higher. Of the precise nature of the more elementary tissues before these observers nothing scarcely was known, and of the true condition of their secondary modifications very inexact knowledge existed. The History of the Anatomy of Tissues formed but an insignificant portion of the botanic treatises of the day, and too often the products of supposition were given to the reader in lieu of the results of observation and experiment. One great reason of this, undoubtedly was the comparative infrequency of the use of the Achromatic Microscope, by which instrument alone, when high powers are employed, can that exact definition, sufficiency of light and comfort and ease of observation, so as to permit, or induce to prolonged and careful investigation, be at one and the same time obtained. With the aid of this important and valuable instrument, guided by the more close and rigorous system of reasoning, which has overruled the science of general physiology in modern times, Meyen, Mohl, Link and many others, have erected that superstructure, which must be allowed to have bestowed upon this department of botany the form, character and exactitude of its analogue in the history of animals and man. Upon the important and fundamental subject of cell-development we would refer the reader to an elaborate Memoir by Carl Nägelli, published in the volume of "Reports and Papers on Botany" of the Ray Society for 1846, as also to the Section on "The Cell as an Individual," in Mr. Henfrey's Outlines, in which also a review of Nägelli's own views will be met with. The Reports of Link, and the Jahresbericht of Meyen will likewise be found to be the best summaries of what has been effected in our enquiries into the general internal structure of plants.

To Kützing, Decaisne, Montagne, Berkeley, Hassall, &c. &c. we are indebted for much exact information upon the structure of the lower tribes, or the so-called *asexual* plants, which, from their minuteness and obscurity, the botanists of earlier times were too willing to leave alone. In connexion with these unassuming organisms there have arisen some curious and important questions in relation to the physiology and diseases of man and animals, and also a revivification of a doctrine of metamorphosis taught in the schools of philosophy of old. It has been asserted that, many diseases of man and animals have, as their essence, the development of minute parasitical fungi and confervoid plants upon and in the living form, and since the paper of Hanover, "On a Contagious Conferva growing upon the Water Salamander," in Müller's Archives for 1839, the in-investigation of the subject has been prosecuted both at home and abroad with considerable success towards the establishment of some new principles in pathology. Of these, the chief are, first, that the morbid lesional essence of certain affections is in the development of the vegetable parasitic organisms above alluded to, and secondly, that—'the contagious character of many diseases is owing to the direct transmission of morbific cells, which seem to possess a semi-individual life, from one person or animal to another;' and that it is impossible to draw a line of demarcation between these primordial cells of the vegetable and animal kingdoms which propagate themselves in one and the same mode. 'Each cell enjoys as it were a separate individuality, so long as it is supplied with nourishment prepared for its assimilation, and effects the multiplication of its kind by the development of new cells of its own nature within itself.'—'There can be little doubt that a cancerous tumour of any size may be developed from a single cell, and it seems very difficult to draw the line which shall separate such independent growths, on the one hand, from the ordinary tissues of the body (in their primordial condition), and, on the other, from structures really parasitic."—(*Carpenter's Animal Physiology*).

With respect to the vegetations which occur in and upon living bodies, we must refer the reader to Link's Report for 1842-43, as also to the work of Klencke—*Neuen Physiologischen Abhandlungen, Leipsic*, 1843.

Bearing somewhat upon this subject, the Memoir of Unger on Plants observed at the moment of their transition into animals, *Vien*. 1843, and the notices of treatises having a like relation referred to in Link's Report for 1842-43, will be of service to the student. Great value has resulted to Botany from the attention bestowed by Schleiden, Mohl, and Lindley, upon the structures and growth of the stems of Endogens, a tribe of plants most interesting to man, as affording him more food and fewer poisonous species in proportion to their whole number than Exogens do. To Schultz and Lindley also, we are indebted for valuable information upon the same points connected with last-mentioned division of plants, and are led to believe, from their endeavours, that the period is not very far distant when the structure of the stem will be much more investigated than it is at present, and be employed for the foundation of good and important divisions in Systematic Botany.

Much also has been effected, amongst other things, in relation to the nature and structure of the organs of reproduction, from the discovery of the *Antheridia* and *Pistillidia* of the muscales up to the elaborate investigations of Griffith, Schleiden, and Decaisne, of the generative apparatus of Santalum and Loranthus. In the opinion of some, our knowledge of the reproductive organs of the muscal alliances is so exact, that they do not hesitate with Montagne in asserting that "nobody now-a-days doubts that mosses and liverworts have two sexes." But though much has been determined upon and proved with respect to this point, formerly so dark a subject with the older botanists, we are scarcely as yet warranted in believing this statement, and must accord, with Dr. Lindley, that the question of sexes in the muscal alliances is *undecided.* There is no doubt that two very different sorts of organs exist among their species, but we have not at present sufficient evidence to shew that the *Antheridia* are analogous to the stamens of the higher and more perfect orders. Mr. Griffith, however, in a very able memoir on the genera Azolla and Salvinia, published in the Calcutta Journal of Natural History, agrees in this belief of the sexuality of Acrogens. But, as has been observed, the question is not whether there may not be, in such plants as these, some trace of a male and female principle, or certain organs in which it is probable that such a principle resides, but whether there is any structure as that which we know to be sexual in the plants of the upper families.

Whilst noticing the advances made in our knowledge of the sexual apparatus of plants, we cannot help directing the attention of the reader to the valuable work of Dr. Lindley, entitled "Illustrations of the Genera and Species of Orchideous Plants." In this beautiful book it will be seen how different are the views now taken to formerly of the generative organs of this strange and highly remarkable group of plants. Although as early as 1810 Robert Brown, in his Prodromus, and after him Richard, placed before botanists a rational explanation of the structures in question, yet, in the work alluded to, we are first made acquainted with the labours of one now removed from us by death, which prove, beyond a doubt, that although we were not aware of the results of his labours until the year 1830, yet, before the commencement of the present century, he was master of much of the subject in question.

"But long before the publication of any rational explanation of the structure of orchids, while botanists were in utter darkness upon the subject, it had been investigated by a man unrivalled in his day for the perfection of his microscopical analysis, the beauty of his drawings, and the admirable skill with which he followed Nature in her most secret workings; and, let me add, which is a still rarer quality, the generous disinterestedness with which he communicated to his friends the result of his patient and silent labours. Sketches were executed by the late Francis Bauer, between 1794 and 1807, in which the most material part of what has been published since that period is distinctly shown, and it has been my good fortune to be the humble means of giving some of these remarkable productions of the pencil to the world in the illustrations of the genera and species of Orchidaceous plants."

Although it was as early as 1790 that the German poet Goethe made known his views on *Vegetable Metamorphosis,* faint glimmerings of which had been possessed by Wolff, yet we are indebted to De Candolle, Turpin, Lindley, Martius, and others, for a rational exposition of this most important branch of the science of Botany. The principles of Morphology have, by the labours of these writers, made clear and satisfactory that which before was "a tissue of ingenious misconceptions," and have given a rational explanation of that which, without being tested by their application, we never could have known—viz. the History of the Fruit. As a condensed but precise view of the nature and general theory of vegetable metamorphosis, we can recommend the short chapter upon this subject in Dr. Willshire's "Principles." The views there offered to the student are founded upon the writings of the German Botanist Von Martius. It is but fair to own, however, that great as the value of the doctrines of morphology is, and more especially in their application to explain the nature of the Ovarium and Fruit, yet that we accord with Dr. Willshire in his statement, that in some cases these doctrines have been carried too far, so much stress being laid upon analysing down to preconceived types, that the true nature of the existing synthesis has been often misunderstood or disregarded. Whilst speaking of the Fruit, we would express our opinion that a great benefit has been conferred by Dr. Lindley upon Botany and its student, by the admirable carpological arrangement given in his "Introduction," which is made primarily to depend upon the structure of the ovary, by which the fruit is of necessity influenced in a greater degree than by anything else, the fruit itself being in fact but the ovary matured, although, as Dr. Lindley remarks, the abortion and obliteration to which almost every part of the ovarium is more or less subject, often disguises it to such a degree, that the most acute carpologist would be unable to determine its structure from an examination of it in a ripe state only. Of the value of the principles of morphology, the student will find also the memoir of Dr. Zuccarini, on the Morphology of the Coniferæ, translated by Mr. Busk, in the "Reports and Papers" of the Ray Society, an admirable and instructive example.

Leaving structure and its metamorphosis, and casting our eyes upon the history of function, and what relates to the theory of the general nutrition of plants, we shall find that much also has been done. The physiology of vegetable organisms has acquired a new phase, each separate action of vitality has been analysed and examined, apparently to the most ultimate law we can attain to. The process of germination and the movement of fluids in plants, the functions of digestion, respiration, and secretion, the development of colour, and the generation of heat and light, the nature of, and amount of irritability possessed by vegetables, their growth and propagation, have all been by modern physiologists minutely investigated. Among the numerous works illustrative of these subjects, we cannot refer to a better systematic guide than to the elaborate book of Meyen on "Pflanzen Physiologie," in which will be found how much we *now* know, and a history of what was known before.

Upon the investigation of the processes of impregnation, fertilization and development, the amount of labour which has been expended by the most acute physiologists is enormous. The remarkable doctrine of Schleiden—that we have hitherto named the sexes in plants quite wrong, for the grain of pollen must be looked upon as giving the form of the new being, and therefore the anther is a *female* organ, and the sac of the embryo is to be regarded as the *male* principle, but which only dynamically determines the organisation of the material production, (*Willshire's Principles*) —has been to a great extent supported by Wydler of Berne, who remarks—that plants have not their sexes disposed in the manner hitherto believed—the anther, so far from being a male organ, is a female one—it is an ovary—the grain of pollen becoming the embryo—(*Willshire, op. cit.*) The enunciation of the above views has caused, as may readily be imagined, much discussion upon this obscure function of the living being, and although they have not met with general acceptation, yet great increase of knowledge has been the result of the enquiry.

Upon the subject of generation we must refer also to the views of Endlicher and Mirbel, to Decaisne on the Fertilization of the Miseltoe, to Hartig's " New Theory, &c." and to the notices of several memoirs in the volume of the Ray Society for 1845. The Third Volume of Meyen's Physiology also, may be consulted with great advantage.

Upon the curious and interesting subject of the development of heat and light by plants, the last-mentioned work may be referred to. This has been but little considered by our own writers, but we believe Dr. Willshire was the first who, borrowing from Meyen, gave it a separate consideration in the History of the Functions of Plants.—(*Willshire's Principles,* page 92, et seq.)

We cannot allow Hartingh's paper on the Growth of Plants, Daubeney's Lecture on Agriculture, and Liebig's Organic Chemistry in its application to Agriculture and Physiology, to pass without notice, as having all had considerable influence in raising the physiology of plants to the point it has attained.

Of the advances made in the systematic portion of the science, no better proof can be offered than the work of Dr. Lindley, to which we shall immediately direct the special attention of the reader, and as affording it a judicious analysis of what others have done, we can refer to it with pleasure. Speaking of the " schemes" of various degrees of merit, some of which have dropped still-born from the press, while others continue to enjoy well-deserved reputations, Dr. Lindley remarks :

" It would be alike unjust to their authors and the public to omit all mention of even the most obscure of these, each of which has been the result of much thought and patient study, and has doubtless contributed something to the progress of systematic science. But it would be beyond the objects of the present sketch to treat them all at length, nor would the student derive any advantage from doing so; while therefore the following pages will be occupied by some account of every plan for a natural classification of which I have any knowledge, since the year 1789 inclusive, and of those of Ray and Laennec of an earlier date, such as are comparatively unimportant will be dismissed in a few words, and those only which have been really employed in practice will be stated at length."

Of course the notions of the German Metaphysical Natur-philosophists, such as Oken, &c. are not held necessary to be considered.

Overruling this department of botanical science, a peculiar doctrine has emanated from a very high source which, if true, would cause great revolution in all present systems of arrangement. M. Alphonse De Candolle, contrary to the axioms of Ray (Hist. Plant. Vol. I. Pref.) and of Linnæus (Phil. Bot. 77), requires that *absolute limits* should be assigned to all groups, of whatever degree. If, says this writer, we cannot state in what respect two families differ permanently and universally, then two families are but one. Two pieces of land which touch each other form one island, and not two, but two pieces of land which are separated by an arm of the sea form two islands, and not one (Annales des Sciences Nat. Ser. 3, Vol. I. p. 254). But, remarks Dr. Lindley, " this kind of reasoning is wholly inapplicable to natural history, for the reasons so admirably given by Ray, and is contrary to all experience. If the groups limited by M. Alphonse De Candolle himself are examined by this standard, they alone suffice to demonstrate how visionary are such expectations. It would be very convenient to find his views practicable, but in truth they are quite Utopian." We may refer also to Mr. Bentham, in the London Journal of Botany, 4, 232, who " has satisfactorily answered the learned Botanist of Geneva."

On reference to Professor Griesbach's Reports, on the Contributions to Botanical Geography, the amount of the late expenditure of labour upon the subject in question can be fairly judged of, and it is with great satisfaction we can refer to the publication of a translation, by Margaret Johnston, of Meyen's Outlines of the Geography of Plants, by the Ray Society last year. In this interesting work will be found considered the conditions of climate which determine the presence and distribution of plants, and by which the soil influences their station, &c. The Physiognomics of Vegetation, the Statistics of Plants, and the History of Cultivated Species, employed as necessary foods, useful as luxuries, and as used in the manufacture of stuffs and materials indispensable to man, are treated of in detail, and there is prefixed an Introduction to Botanical Geography, with a list of the principal works which have appeared on the subject since Alexander de Humboldt's Essai sur la Geographie des Plantes, accompagnèe d'un Tableau Physique des Regions Equinoxiales, Paris 1805, 4to. We add the following remarks in connexion with the work of Meyen, as the subject is one of considerable interest at the present time.

In 1812 an attempt was made to draw attention to the value of the cultivation of Maize, or Indian Corn, the Zea Mays of Botanists, by Parmentier (Le Maïs ou Blé de Turque a Paris 1812), and M. F. de Neufchateau followed with a Supplement to the above work in 1817. Within the last few months, the same endeavours have again been made, and hopes have been held out that the cultivation amongst us of this cereal grass may in future diminish the risks attendant upon the exclusive cultivation of the potatoe, and the cereals usually grown. How far this is true is doubtful. According to some there is a particular line on the Continent of Europe, north of which maize will not thrive. It will not ripen its grains on poor land or without attentive cultivation, the land must be naturally fertile or made so by art, and, as it could not be safely sown in England before the middle or end of May, in many seasons it could scarcely be expected to ripen its seeds before the winter frost set in. The time of flowering is said also to be very critical to the maize plant, a cold damp atmosphere often causing a great part of the crop to fail, and of all the cereals which are grown by various nations, none except the rice is so unequal in its products. It is stated by Baron Humboldt, that on the same soil, the produce of a grain will vary from 40 to 200 or 300 fold, according to the variation of the moisture and mean temperature of the year, and its culture has the advantage and disadvantages of the vine.—Meyen remarks—

" Maize thrives best in the hottest and dampest tropical climates; there are some places where it brings forth 800 fold; in less fertile lands, the produce is 300 or 400 fold, and 100 fold is looked on as a poor crop of the grain in tropical climates. Maize is less productive in the temperate zone, the average yield in California, between the latitudes 33° and 38° is not greater than 70 fold. In yet colder countries the yield is still smaller, and our cereals gradually supersede the maize, this is the case in Chili, where maize is grown as a vegetable only, and wheat is used as bread. We do not know the exact polar limits of maize culture in the New World yet, so much is certain that they lie in the 40th parallel; even in the southern hemisphere where, particularly in Chili, from many causes, the climate is much lower in proportion to the latitude, the maize is cultivated as low as 40°. On the western coast of Europe maize is grown in 45½ north latitude; on the Rhine to 49°, and in our country even in 52°. Large and abundant crops are raised in gardens, yet with us there is little taste for this fine grain, and therefore its culture is neglected. Maize is only grown to adorn our gardens, and the rich produce is given to cattle in Germany. Maize is most extensively cultivated in the rich valley of the Rhine, known by the name of the Bergstrasse, but this district is also the warmest in all Germany.

"At the present day maize is cultivated in all the countries of the tropical and temperate zones which European civilization has reached; it cannot, however, supersede the culture of the earlier cultivated cereals."

Dr. Lindley and Sir W. T. Hooker have done more for the advancement of botanical science in this country amongst the general body of naturalists, than has been effected by the conjoint labours of all other men. The former, by his able writings on its great principles, whether applied to the structure, function, or the systematic grouping of plants, as also by his efforts as an oral preceptor; the latter, by the valuable aids he has afforded to the practical botanist by his minute investigations into the Flora of our own land, and the production of the best work on the numerous species which flourish in it. We have other great names it is true, and men whose reputations are deservedly spread far and wide. But they have not had the fortune to be able, or the inclination to appeal so much to the popular and every-day requisitions of the mass, as those we have particularly alluded to. *They* have done so, and by their labours have not only met the exigencies which existed, but at the same time have almost reorganised the aspect of botanical science, have bestowed a new phase upon their favourite study, widely extended its boundaries, endowing it with a comprehensive purpose and philosophic character meeting with the general acceptance of the best authorities of the present day.

With the public at large the study of and the patronage given to the investigation of the wonders of the vegetable world must be, by the most enthusiastic of admirers, confessed to be extensively bestowed. Our botanic and horticultural gardens, our flower-shows, our repeated sales of foreign bulbs and plants are proofs of this, together with the numerous works on botany and vegetable physiology for young persons, mainly the results of the labours of delicate and feminine hands. To Mrs. Loudon in particular we stand much indebted. We have but to call to mind the beautifully illustrated works published by Mr. Lovell Reeve and Mr. Van Voorst to be satisfied how much is expended both by the public and publisher. The student of medicine is now amply supplied with works not only evincing the advanced condition of botanical science, but the desire of adapting it to his special wants and requisitions. The course of lectures upon it, which he is desired by the governing bodies to attend, is a most necessary and valuable introduction to the higher department of the history of the structure and functions of the human body. The names of

Roxburgh, Wallich, Griffiths, Royle, and of others, will prove to him how available in a foreign climate may be the exact botanical information he can, whilst a student at home, so easily obtain, and how bitterly he may rue, perhaps at some future day, the want of that knowledge which at present is so readily at his command.

It has been often urged that the difficulties required to be mastered before the professional student is able to avail himself of the advantages of the *Natural system* in interpreting "the hidden characters with which Nature has labelled all the hosts of species which spring from her teeming bosom" are great in the extreme, and scarcely within the scope of a limited period of study, or that they present more obstacles in overcoming than the fundamental doctrines of other branches of natural science.

"But of that difficulty it may be observed, in the first place, that it is only such as it is always necessary to encounter in all branches of human knowledge, and secondly, that it has been much exaggerated by persons who have written upon the subject without understanding it.

"It has been pretended that the characters of the natural classes of plants are not to be ascertained without much laborious research, and that not a step can be taken until this preliminary difficulty is overcome. But it is hardly necessary to say that, in natural history, many facts which have been originally discovered by minute and laborious research, are subsequently ascertained to be connected with other facts of a more obvious nature, and of this botany offers perhaps the most striking proof that can be adduced.

"It is true that careful observation and multiplied microscopical analyses have taught botanists that certain plants have spiral vessels and others have none, but it is not true that in practice so minute and difficult an enquiry needs to be instituted, because it has also been ascertained that plants which bear flowers have spiral vessels, and that such as have no flowers are usually destitute of spiral vessels, properly so called; so that the inquiry of the student, instead of being directed in the first instance to an obscure but highly curious microscopical fact, is at once arrested by the two most obvious peculiarities of the vegetable kingdom."

If, however, the distinctions of classes and sections in the so-called *artificial systems* are simple and easy to remember, in the determination of more subordinate groups as of genera, the difficulties are far greater by these than by the natural methods now adopted for arranging plants. This fact Jussieu long ago remarked, and shewed that, whatever trouble is experienced in remembering or applying the characters of natural orders is more than compensated for by the facility of determining genera, the characters of which are simple in proportion as those of the orders are complicated. But, as Dr. Lindley remarks:—

"All considerations of difficulty ought to be put aside when it is remembered how much more satisfactory are the results to which we are brought by the study of Nature philosophically, than those which can possibly be derived from the most ingenious empirical mode of investigation."

In the work of Dr. Lindley, to which we now especially direct the attention of the reader, we find the results of the most extended practical knowledge of the systematic portion of the science of botany, and of the writings of other labourers, both of this country and the continent. Results developing themselves so admirably in relation to the necessities of the student, whether we regard *what* he should learn or *how* he should learn it, and information of the highest value even to the most advanced in knowledge, made so easily accessible, demand at our hands unqualified praise. In fact, we hesitate not in saying that the publication of the more than 900 pages before us will form quite an epoch in the *Bibliographia Botanica* of the day. No pains or expence appear to have been spared in the mechanical production of the work, the illustrations are admirable, often exceedingly picturesque, and the whole book a monument to the credit of one of the most pains-taking of men. The general intention of our author will be seen by the following extract from the Preface :—

"Its object is to give a concise view of the state of systematical Botany at the present day, to show the relation or supposed relation of one group of plants to another, to explain their geographical distribution, and to point out the various uses to which the species are applied in different countries. The names of all known genera with their synonymes are given under each natural order, the numbers of the genera and species are in every case computed from what seems to be the best authority, and complete indices of the multitudes of names embodied in the work are added, so as to enable a Botanist to know immediately under what natural order a given genus is stationed or what the uses are to which any species has been applied. Finally, the work is copiously illustrated by wood and glyphographic cuts, and, for the convenience of students, an artificial analysis of the system is placed at the end."

The Introduction of the work is taken up with an able exposition of the various *Natural Systems* proposed by botanists from the time of Ray—1703—to the present time, concluding with the classification proposed and followed by the author himself, together with a review of those principles which should guide us in the formation both of the greater and minor groups of the vegetable world.

The grand object of the author next follows—the consideration in detail of the numerous natural classes into which he divides the 92,000 plants known existing. This portion of the work may be said to consist of two great divisions ; one in which the *Asexual* or flowerless plants are included, the other whose aim is the elucidation of the various tribes of the *Sexual* or flowering ones.

Preceding each subdivision—such as of Thallogens Acrogens, &c. &c. will be found an able *resumé* of the general structure and habits of the plants typical of those of the various groups which follow, and we would particularly draw the attention of our readers to the value of the method adopted to afford at a glance the indication of affinities, which consists in printing at the end of the list of genera the name of the Order under discussion in capital letters ; placing right and left of it, in small Roman letters, the names of those orders which are supposed to be in nearest alliance to it ; and above and below it, in Italic type, the names of such as are only analogous, or at least have a more distant affinity. Dr. Lindley has borrowed this idea from the paper of Mr. Strickland, on the True Method of discovering the Natural System in Zoology and Botany, printed in the *Annals of Natural History, Vol.* VI. *p.* 184.

We entirely agree with Dr. Lindley as to the propriety of omitting any discussions on the proximate principles and ultimate chemical constituents of plants. These are truly questions of pure chemistry and not of botany, have no relation to any known principles of botanical Systematology, and more than-one half of the daily discovered vegetable products are nothing but the *manufactures* of the chemist's laboratory. Should information connected with the subject, however, be required by the botanist, he will find much in the writings of Mohl and Payen, and in the Reports of Dumas, Pelouze, and Adolphe Brogniart, abstracts of which will be found also in the volume of the Ray Society for 1845, in Link's Report on the Progress of Botany. Upon these matters truth obliges us to remark that, it is a stumbling-block in the way of advance, yet needed in this department of science, that men famous in different branches of knowledge should be jealous of each other's reputation. We quote the following remarks of Schleiden from Link's Report above alluded to.

"On reading the good-for-nothing opinions of Berzelius and Liebig respecting Schwann's discoveries with regard to the (Gährungspilz) fungus fermentation, one would suppose that these gentlemen had never heard of such a thing as a microscope. On hearing Berzelius speak of Schwann's frivolousness we do not know what to say to such absurdity. I should sincerely congratulate the science of chemistry if Berzelius had always instituted his researches with a circumspection so thoroughly founded upon elaborate knowledge, and a profundity combined with so much modest doubt in his own powers as to screen him from the influence of preconceived opinions as Schwann has done. Did not the first hundred pages of the sixth volume of his Chemistry occur to Berzelius when writing these words, and produce a blush of shame in him for such a judgment?"—(*Dr. Lancaster's Translation.*)

Whilst so freely according the most merited praise to the author of the *Vegetable Kingdom*, we must unite with those who disagree with him in the attempt to introduce the fashion of Anglicising Latin and Greek names in the manner he seems to have so much at heart. We deny that it is an available or a useful project, the more especially so in a work confessedly scientific, as the one before us must be held to be, however much it is intended at the same time, to diffuse a knowledge of plants throughout general society. Some of our *scientific* names, undoubtedly, are bad enough, and made up of bits from all sorts of languages—witness such terms as *Kraschenninikovia, Andrezijofskia,* and others; but, as a general rule, they are quite as euphonious as those proposed by Dr. Lindley for the English scholar, and more acceptable to the systematic botanist. We find nothing very mellifluous about such words as *Helwingiads*, *Turnerads*, *Artocarpads*, or *Cedrelads*; in fact, we hold *Turneraceæ*, *Artocarpaceæ*, and *Cedrelaceæ* to be infinitely their superiors—so also we prefer *Algaceæ* for *Algals*, and others we could name.

The student of medicine will find ample description of the uses and properties of the more important plants, which are arranged under the various *natural families*, described in the work before us, and considerable research appears to have been bestowed upon this portion of our author's labours. Occasionally, however, we meet with something which makes the professional reader smile—such as

"*Solanum dulcamara*, the bitter-sweet, is a strong narcotic in its foliage, and its berries are by no means safe, although it does appear that in some cases they have been taken into the stomach without inconvenience.—*Solanum nigrum*, a very common weed in all parts of the world, except the coldest, is more active. A grain or two of the dried leaf has sometimes been given to promote the various secretions, *probably by exciting a great and rather dangerous agitation in the viscera.*"

At page 620 it is stated that *Tobacco* is a *powerful stimulant* narcotic—we think it very doubtful if tobacco has the least stimulant virtue whatever, except where it is immediately and locally applied—on the contrary, we believe that it is one of the most *direct* sedatives known.

*Cicuta virosa* is described as producing effects similar to those of Hydrocyanic acid, and of which we have also great doubts. It is amusing likewise to find the numerous virtues and powers which have been ascribed to many members of the vegetable kingdom, some of which, from the accounts which have been given of them, might be thought to be powerful rivals to the famed *Ginseng* of the Chinese, the *Panax quinquefolium* of systematic writers, and upon which Pere Jartroux says, that the most celebrated physicians of China have written volumes, affirming it to be able to ward off or to remove fatigue, to invigorate the enfeebled frame, to restore the exhausted animal powers, to make old people young, and, in a word, to render man immortal (this saving clause being however added), "if any thing on earth can do so." This wonder of the world, this dose for immortality, has been known to cost its weight in gold. For the "alexipharmic," "cephalic," "corroborative," and "aphrodisiac," &c. virtues of these vegetable miracles, our profession, however, and not Dr. Lindley, are bound to produce proof. Connected with the properties of plants, we are tempted to give the following extract from our author, to shew the fund of information which is to be obtained from his work, and to correct what we believe to be a very general error of opinion, that the natural family *Fabaceæ* or *Leguminoseæ*, only affords plants possessing little or no active or deleterious power when taken into the body.

"In a very large number of species narcotic properties have been recognized. The seeds of Lathyrus aphaca have been already mentioned. Those of Abrus precatorius, whose scarlet seeds with a black scar are commonly used as beads, Anagyris fœtida and others, have a similar property. This is, however, positively denied in the case of Abrus, by Dr. Macfadgen (? Maclagen), who asserts them to be harmless, and merely indigestible. The leaves of Arthrolobium scorpioides are capable of being employed as vesicatories. The juice of Coronilla varia is poisonous. The roots of Phaseolus radiatus are narcotic, and so are those of P. multiflorus, the scarlet running kidney bean, which a year or two ago poisoned some children at Chelsea who had partaken of them. Both the Laburnums (Cytisus alpinus and Laburnum) have caused serious accidents to children who have swallowed their venomous seeds, and C. Weldeni is reported to poison the milk of the Dalmatian goats that browse upon its foliage. The dye called Indigo is a formidable vegetable poison. Schomburgh states that the violet blossoms of Sabinea florida are dangerous. The seeds of Ervum Ervilia, the Bitter Vetch, mixed with flour and made into bread, produce weakness of the extremities, and render horses almost paralytic. Andira inermis and retusa, and some Geoffrœas, especially G. vermifuga, and G. spinulosa, have an anthelmintic bark, with a disagreeable smell, and a sweet mucilaginous taste; the effects are drastic emetic, purgative and narcotic, poisonous in large doses, producing violent vomiting, with fever and delirium. A few years since, hundreds of sheep perished in the Swan River Colony, in consequence of their cropping the leaves of some wild plant there; according to an official report, it was a Burtonia that produced the mischief, but, according to Mr. James Drummond, the mischief was caused by a Gompholobium. Nothing, however, more plainly indicates the venomous nature of Leguminous plants than their being used as fish poisons. The bark of the root of Pisidia Erythrina, a common Jamaica tree, is a very usual fish poison in Jamaica, and yields a most remarkably narcotic and diaphoretic tincture. Many Tephrosias are employed in the same way, especially T. toxicaria, the young branches of which, with the leaves pounded and sometimes mixed with quick-lime, are thrown into a pond of some mountain stream, and have an almost immediate effect. The fish are observed to become stupefied, and as it were intoxicated, and to rise to the surface, floating there with their belly upwards, so as to be readily taken by the hand. It has been remarked that the larger fish recover gradually from the effects of the poison, but that the younger fry perish. It has been suggested that, the action of the plant upon the human system would resemble that of Digitalis, and might prove, in a climate where that plant does not grow, a desirable substitute." P. 548.

Of Mr. Henfrey's work, we have as yet received but the first two parts—a third and concluding part having to follow. Unlike the work of Dr. Lindley, the great aim of which is to develop the characters and affinities of systematic groups of plants, this, of Mr. Henfrey, has for its endeavours an exposition of the general and special anatomy of vegetable bodies and the history of their functions. Much of the former of these subjects is of course found in the "Vegetable Kingdom," but, whilst there it is developed *analytically*, here it is exhibited *synthetically*.

The first part is occupied with a consideration of elementary structures; the second, with an exposition of the organs of vegetation, and certain of the functions of plants. The chief object of the author being "to give a concise view of the actual state of our knowledge at the present time, to the exclusion of all hypotheses hazarded without sufficient ground or negatived by experience, the various points are treated as they rise progressively in complexity; by this means the development and morphology of structures and organs will be more easily explained, and at the same time will conduce to the simplification of the subject, by leading to the recognition of an unity of plan throughout the vegetable kingdom."

We readily bestow upon the author the merit of having furnished the student with a work more in accordance with the advanced state of the science of vegetable anatomy and physiology, as it exists in France and Germany, than any which he before possessed; and if no great originality, either of text or of illustration, is to be met with in his book, yet in it will be found a simple analysis of the writings of *Mohl, Schleiden, Meyen*, and others. There is great amount of information compressed into a narrow compass along with ample illustrations, though the latter, in our opinion, greatly differ from each other in merit of execution.

We must object to what appears to us a frequently loose application of terms met with in Mr. Henfrey's work—why, for instance, are such things as *porous cells* spoken of when no such structures exist? It is true that Mirbel, some years ago, spoke of cells, "the sides of which are riddled full of holes," but this has long been shown to be an error, and the true nature of such *pitted* tissue properly demonstrated. Even Mr. Henfrey is afterwards obliged to modify his statement, admitting that, in the *normal condition* of the tissue in question, there is no solution of continuity of the primary membrane forming the wall of the cell, and that, consequently, no hole or *pore* can exist.

"In old cells the primary membrane has become absorbed, and the space between the rings, convolutions, or reticulations, are thus converted into holes. *It is probable that this does not take place until after all deposition has ceased, and the cells in such case may be considered as dead.*" P. 25.

" In their original condition they are *always closed* by the primary membrane, and their contents are only in relation by endosmosis." P. 27.

It may be our own fault, but we must confess we do not understand the following definition of one kind of our author's *porous cells.*

" B. Cells with bordered pores, arising from the presence of a lenticular cavity between two adjacent pores." P. 25—26.

In our opinion, the distinction between certain forms of underground stems and roots is not made by any means sufficiently clear to the student. At page 65, the turnip is alluded to as a *root*, whereas, from the researches of Turpin, there is no room to doubt that the turnip, the radish, the cyclamen, and the elephant-foot, are all distentions of the stem, either of the first internodium, or of the inferior prolongation of the stem below the cotyledons, and above the true root.—*Vide Lindley's Introduction to Botany.*

Again, a *tuber* and a *tuberous root* are essentially different in their nature, yet Mr. Henfrey speaks of them as one and the same thing.

" These enlargements are called tubers, and the roots are said to be tuberous." P. 65.

At page 93, however, it is remarked—

" There is occasionally some difficulty in determining the point, as in such subterraneous branches as those which form the tubers of the potatoe. The expansion of this branch here closely resembles the expansion of the roots in the tubers of the dahlia, &c. The eyes, as they are called, are however true buds, which develop branches above ground, while the aerial buds may be made to form tubers by burying them in the ground."

Now, we affirm there is not the least difficulty in determining whether the tuber of the potatoe be a root or a spheroidally developed subterranean stem, and the only analogy is in *form* between the potatoe and the dahlia. The development of buds by the tuber of the potatoe is at once decisive of its true nature. Mr. Henfrey applies both the terms *nerves* and *veins* to the reticulations of the fibro-vascular system of the leaf.

" The palmate *nervation* occurs sometimes as in *adiantum,* but the pinnate is the more common The terminal divisions of the *veins,* however, in either case, are bifurcations." P. 79.

At page 73 we meet with the term *median nerve.* This, to the student of medicine, is utterly destructive of the help otherwise derivable from bestowing the same name only on an organ or structure in one kingdom of nature which is like to, or performs the same offices as the organ bearing the like name does in another. There is nothing worse than this delusive application of terms, unless it be " the reprehensible practice of giving two names conveying different ideas to the same organ in the same kingdom of nature." The language of Linnæus cannot be always that of the present day, and we advise Mr. Henfrey to follow the terms and their application made use of by Dr. Lindley.

Our author speaks of *solid bulbs.* Dr. Lindley (Introduction), and we agree with him, says—a solid bulb has no existence. It is a contradiction in terms, unless the so-called solid bulb—the *cormus* of modern writers—be a bud, the scales of which have become consolidated. But, as this hypothesis leads to the very inadmissible conclusion "that, as the cormus of a crocus is essentially the same, except in size and situation, as the stem of a palm, the stem of a palm must be a solid bulb also, which is absurd."

Mr. Henfrey defines the bulb to be a *form of stem.* The bulb is not a stem at all—it is an *appendage* of a stem, or a subterranean bud, destitute of the latter.

We had noted some other passages for comment, but enough has been remarked upon to prove to Mr. Henfrey that he may yet add to the value which his little book undoubtedly possesses.

